# Influence of genetic co‐mutation on chemotherapeutic outcome in *NPM1*‐mutated and *FLT3‐ITD* wild‐type AML patients

**DOI:** 10.1002/cam4.70102

**Published:** 2024-08-09

**Authors:** Quan Wu, Yujiao Zhang, Baoyi Yuan, Yun Huang, Ling Jiang, Fang Liu, Ping Yan, Jiaying Cheng, Zhiquan Long, Xuejie Jiang

**Affiliations:** ^1^ Department of Hematology, Nanfang Hospital Southern Medical University Guangzhou Guangdong China

**Keywords:** acute myeloid leukemia, chemotherapy, molecular genetics, survival

## Abstract

**Background:**

Nucleophosmin 1 (*NPM1*) gene‐mutated acute myeloid leukemia (*NPM1* ^mut^ AML) is classified as a subtype with a favorable prognosis. However, some patients fail to achieve a complete remission or relapse after intensified chemotherapy. Genetic abnormalities in concomitant mutations contribute to heterogeneous prognosis of *NPM1* ^mut^ AML patients.

**Methods:**

In this study, 91 *NPM1*‐mutated and *FLT3‐ITD* wild‐type (*NPM1* ^mut^ */FLT3‐ITD* ^wt^) AML patients with intermediate‐risk karyotype were enrolled to analyze the impact of common genetic co‐mutations on chemotherapeutic outcome.

**Results:**

Our data revealed that *TET1/2* (52/91, 57.1%) was the most prevalent co‐mutation in *NPM1* ^mut^ AML patients, followed by *IDH1/2* (36/91, 39.6%), *DNMT3A* (35/91, 38.5%), myelodysplastic syndrome related genes (MDS‐related genes) (*ASXL1*, *BCOR*, *EZH2*, *RUNX1*, *SF3B1*, *SRSF2*, *STAG2*, *U2AF1* and *ZRSR2* genes) (35/91, 38.5%), *FLT3‐TKD* (27/91, 29.7%) and *GATA2* (13/91, 14.3%) mutations. Patients with *TET1/2* ^mut^ exhibited significantly worse relapse‐free survival (RFS) (median, 28.7 vs. not reached (NR) months; *p* = 0.0382) compared to patients with *TET1/2* ^wt^, while no significant difference was observed in overall survival (OS) (median, NR vs. NR; *p* = 0.3035). *GATA2* ^mut^ subtype was associated with inferior OS (median, 28 vs. NR months; *p* < 0.0010) and RFS (median, 24 vs. NR months; *p* = 0.0224) compared to *GATA2* ^wt^. By multivariate analysis, *GATA2* ^mut^ and MDS‐related genes^mut^ were independently associated with worse survival.

**Conclusion:**

Mutations in *TET1/2*, *GATA2* and MDS‐related genes were found to significantly influence the chemotherapeutic outcome of patients with *NPM1* ^mut^ AML. The findings of our study have significant clinical implications for identifying patients who have an adverse response to frontline chemotherapy and provide a novel reference for further prognostic stratification of *NPM1* ^mut^ */FLT3‐ITD* ^wt^ AML patients.

## Graphical abstract

Our retrospective study confirmed that mutations in *TET1/2,* *GATA2* and MDS‐related genes were related to poor therapeutic outcomes in patients with *NPM1* mutated AML. These findings provided valuable insight for refining risk stratification and optimizing therapeutic strategies for individual with *NPM1* mutated AML.

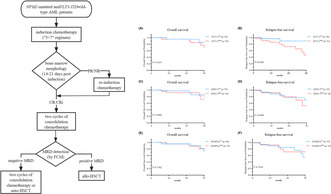

## 1 INTRODUCTION

Nucleophosmin 1 (*NPM1*) gene encodes a nucleocytoplasmic shuttling protein that plays a crucial role in maintaining genomic stability, facilitating ribosome biogenesis, responding to stress, and suppressing growth.^1^, ^2^, ^3^ *NPM1* mutation is more frequently observed in adult acute myeloid leukemia (AML) with a normal karyotype, occurring in 20%–30% of AML patients.^4^, ^5^, ^6^, ^7^ The most common *NPM1* mutation is a 4 base‐pair insertion in exon 12 of the C‐terminal. The base‐pair insertion causes frameshifts and generates a novel C‐terminal nuclear export signal, which is responsible for cytoplasmic localization of mutant NPM1 protein, while the wild‐type NPM1 predominantly localizes within the nucleolus. The aberrantly localized NPM1 had the capability to inhibit caspase‐6/‐8 mediated myeloid differentiation. Moreover, NPM1 mutant disrupts nuclear localization of multiple proteins associated with DNA repair, apoptosis, and differentiation, and maintains homeobox/myeloid ecotropic virus insertion site 1 (*HOX*/*MEIS1*) expression through binding to chromatin loci.^3^, ^8^, ^9^, ^10^, ^11^

*NPM1*‐mutated (*NPM1* ^mut^) AML was recognized as an independent category with a favorable prognosis, based on the recently updated risk stratification by the European LeukemiaNet. Additionally, patients harboring both *NPM1* and internal tandem duplication of *FLT3* (*FLT3‐ITD*) mutations were classified into intermediate‐risk group, irrespective of the allelic ratio.^12^ The *NPM1*‐mutated and *FLT3‐ITD* wild‐type (*NPM1* ^mut^/*FLT3‐ITD* ^wt^) patients had clinically heterogeneity, with approximately 50% of patients died of progressive disease.^4^, ^7^, ^13^, ^14^ The role of genetics co‐mutation was essential in deciphering the heterogeneity of *NPM1* ^mut^ AML patients. Thus, it is necessary to investigate the correlation between genetic co‐mutation and clinical outcome in *NPM1* ^mut^ AML.

The prognostic impact of certain genetic co‐mutations, including *TET1/2*, *IDH1/2*, *DNMT3A*, *FLT3‐TKD*, *GATA2*, and MDS‐related genes in *NPM1* ^mut^ AML patients is still not fully understood, and conflicting results have been reported in recent studies.^15^ We conducted a retrospective study to investigate the influence of commonly coexisting mutations on chemotherapeutic outcomes in patients with intermediate‐risk karyotype and *NPM1* ^mut^/*FLT3‐ITD* ^wt^ AML. Our results demonstrated that *GATA2* ^mut^ was associated with poor overall survival (OS) and relapse‐free survival (RFS), *TET1/2* ^mut^ was related to inferior RFS but not OS. In multivariable Cox regression analysis, *GATA2* ^mut^ and MDS‐related genes^mut^ were independent poor prognostic markers in *NPM1* ^mut^/*FLT3‐ITD* ^wt^ AML patients.

## 2 METHODS

### 2.1 Patients

**FIGURE 1:**
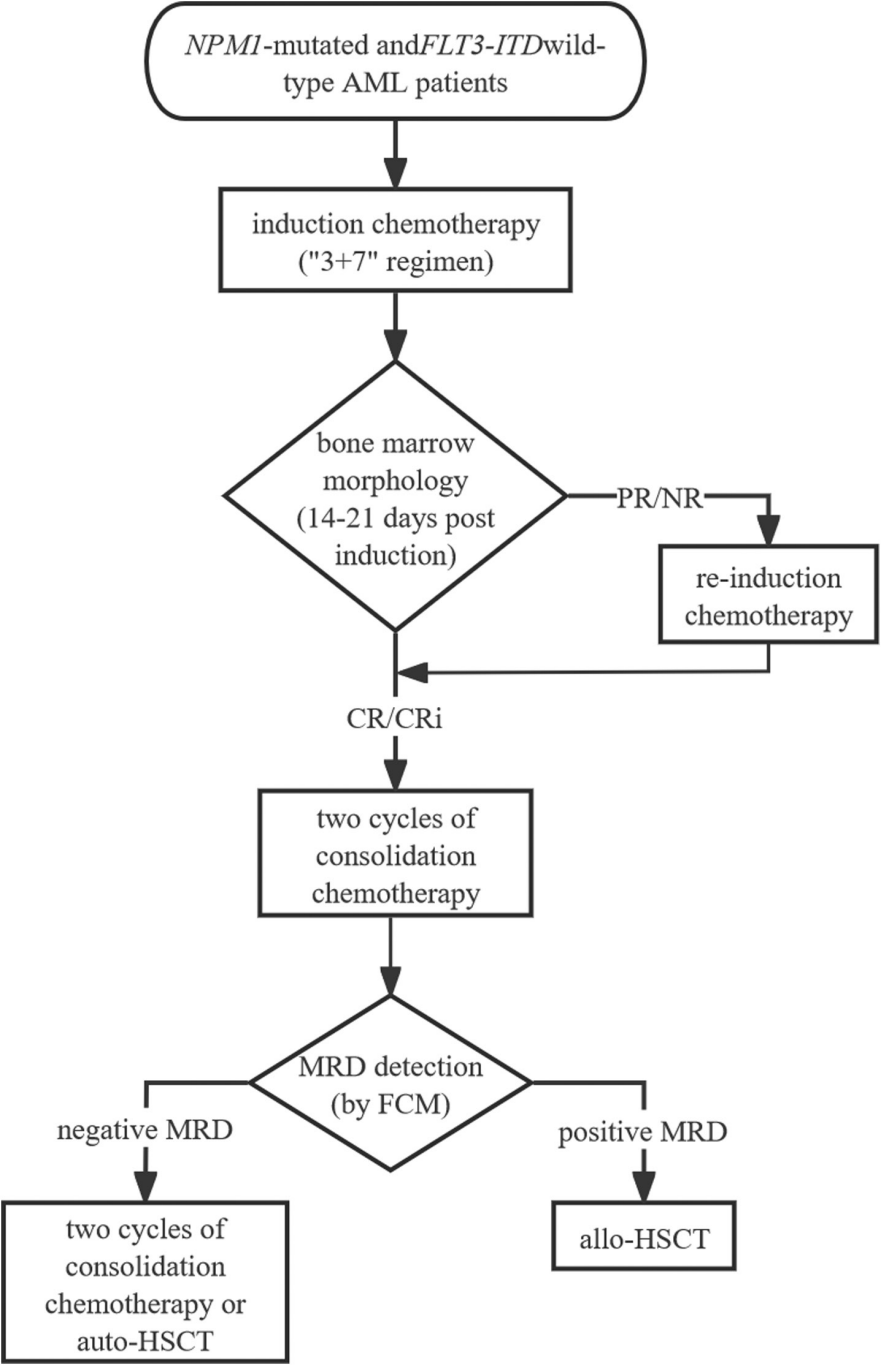
Treatment strategy of the patients. Patients received induction chemotherapy based on “3 + 7” regimen (idarubicin 10 mg/m^2^ or daunorubicin 60 mg/m^2^, days 1–3; cytarabine 100 mg/m^2^, days 1–7). The treatment response was assessed through bone marrow morphology 14–21 days after induction. Patients who achieved complete remission (CR/CRi) after induction received two cycles of consolidation chemotherapy based on high dose of cytarabine (HD‐Ara‐C 2 g/m^2^, days 1–3). Those without CR/CRi received re‐induction chemotherapy (HD‐Ara‐C 2 g/m^2^ plus cladribine 5 mg/m^2^, days 1–5 and G‐CSF 300 μg, days 0–5). Subsequent treatment strategies were determined based on MRD status after two cycles of consolidation chemotherapy. Patients with negative MRD received two additional cycles of consolidation chemotherapy or auto‐HSCT, while those with positive MRD were suggested to undergo allo‐HSCT, unless they lacked HLA‐matched donors or declined transplantation.

In this study, newly diagnosed *NPM1* ^mut^ */FLT3‐ITD* ^wt^ AML patients with intermediate‐risk karyotype aged from 18 to 73 years old were enrolled in Nanfang Hospital (Guangzhou, China) from 2017 to 2020.^16^ The exclusion criteria were as follows: (1) individuals under the age of 18 years, (2) patients with acute promyelocytic leukemia and secondary/transformed leukemia, (3) other carcinomas or severe organ dysfunction at diagnosis, (4) data is not available. The treatment strategy administered to the patients is illustrated in Figure 1. All patients were followed up 30 months for survival analysis. Patients who have undergone hematopoietic stem cell transplantation (HSCT) were considered lost to follow‐up from the date of transplantation.

### 2.2 Molecular mutation detection

The single nucleotide variant and insertion–deletion were identified using Next generation sequencing (NGS) with an NGS‐panel, as detailed in Table S1. The bone marrow samples were analyzed by Flow Cytometry (FCM) to monitor Measurable residual disease (MRD) after each cycle of chemotherapy. Residual leukemia cells were marked by Leukemia‐associated immunophenotypes (LAIPs), including CD34p^+^, CD19^+^, CD38^+^, CD81dim^+^, CD58^+^, HLA‐DR^+^, CD123^+^, CD10^−^, CD20^−^, CD22^−^. A positive MRD was defined as abnormal leukemia population ≥0.1% of the total quantified CD45^+^ cells.

### 2.3 Definition of clinical end points

Treatment response was evaluated according to standardized criteria.^12^ The definition of achieved complete remission with or without complete blood count recovery (CR/CRi) was the presence of <5% bone marrow blasts, absence of peripheral blasts, and absence of extramedullary disease, with or without complete blood count recovery. Relapsed disease was defined as the presence of bone marrow blasts ≥5%, progression of extramedullary disease or reappearance of peripheral blasts. The OS was calculated from the date of diagnosis until death or last follow‐up, with censoring at the last follow‐up if no event occurred. The RFS was measured from the date of remission until either the occurrence of relapse, death from any cause, or last follow‐up, with censoring at the last follow‐up if no event occurred. MRD was defined as the presence of residual leukemia cells below the limit of detection through conventional morphologic evaluation after achieving complete remission.

### 2.4 Statistical analyses

The analysis of categorical variables was conducted using either Pearson's chi‐squared test or Fisher's exact test. The Mann–Whitney *U*‐test or Kruskal‐Wallis test was employed for the analysis of continuous variables. Survival data were compared using the log‐rank test and Cox regression analysis (stepwise selection procedure). Since transplantation strategies may have influenced clinical outcomes, survival data were analyzed after censoring at the time of transplantation. Statistical analyses were performed using IBM SPSS 27.0 and GraphPad Prism 9. A two‐sided *p*‐value of <0.05 was considered to indicate statistical significance.

## 3 RESULTS

### 3.1 Patient characteristics

**FIGURE 2:**
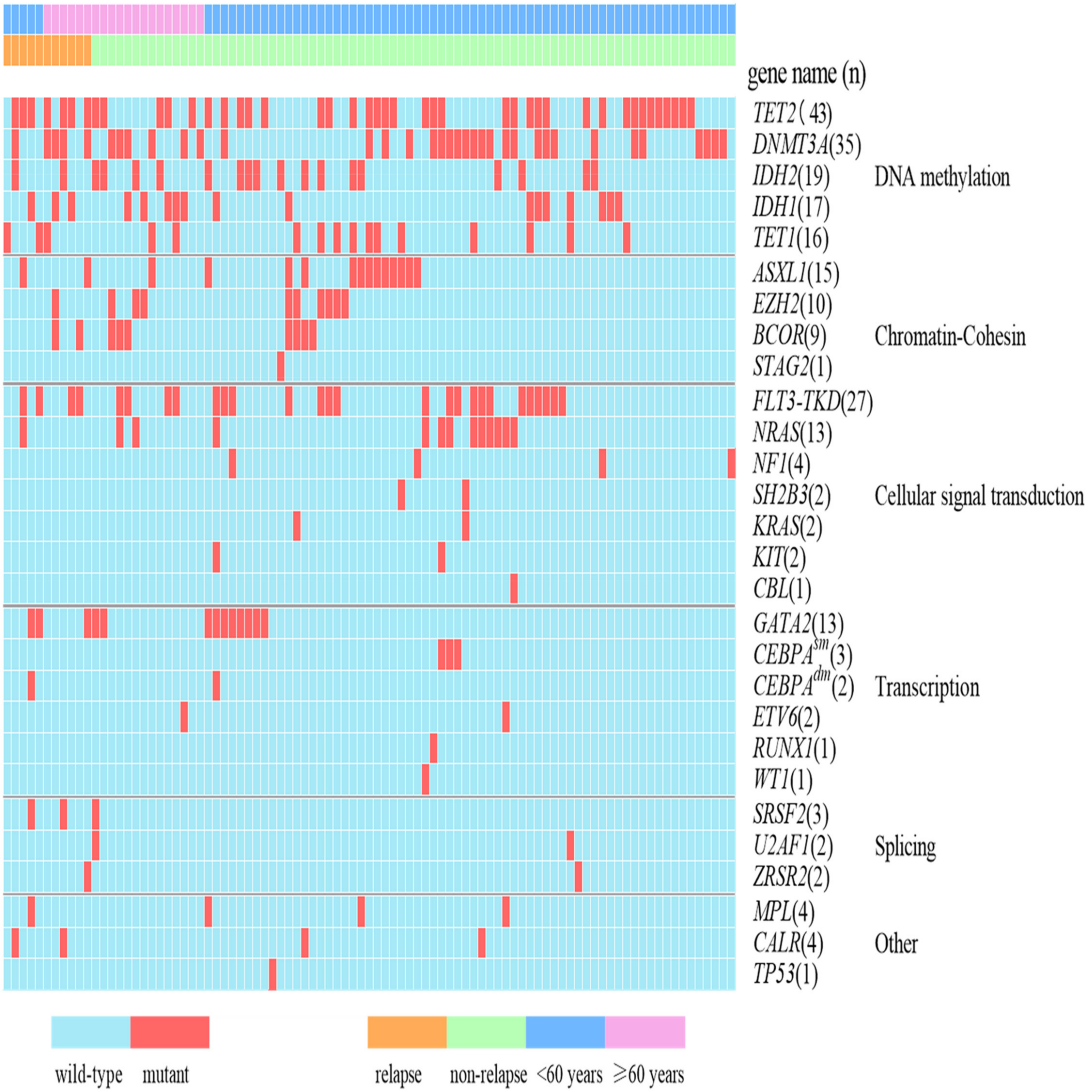
Mutational landscape of patients with *NPM1* ^mut^ at diagnosis. Each column represents an individual case, and each row represents a single gene. Red for mutants, and blue for wild‐types. Mutation in the bZIP region of the *CEBPA* gene was not detected in patients with *CEBPA* ^sm^. *SF3B1* was not detected in all cases.

A total of 91 patients with intermediate‐risk karyotype, characterized by *NPM1* mutation and wild‐type *FLT3‐ITD*, were included in this study. The clinical characteristics of the enrolled patients are summarized in Table 1. The median age of the cohort was 51 years, ranging from 18 to 73 years. The patients did not have complex karyotypes, but minor cytogenetic aberrations were detected in 10 of them (Table S2). In our study, all patients harbored additional mutations, *TET1/2* (52/91, 57.1%) was the most common co‐mutation, followed by *IDH1/2* (36/91, 39.6%), *DNMT3A* (35/91, 38.5%), MDS‐related genes (*ASXL1*, *BCOR*, *EZH2*, *RUNX1*, *SF3B1*, *SRSF2*, *STAG2*, *U2AF1*, and *ZRSR2* genes) (35/91, 38.5%), *FLT3‐TKD* (27/91, 29.7%), and *GATA2* (13/91, 14.3%) mutations (Table 1). The mutation profiles of all patients are illustrated in Figure 2. Notably, none of the patients in our cohort carried *SF3B1* mutation. It was observed that seven patients had both *TET1* and *TET2* mutations, while none harbored mutations in both *IDH1* and *IDH2* (Figure 2). Interestingly, the frequency of *IDH1/2* mutation was significantly higher in older *NPM1* ^mut^ patients (≥60 years) (33.3% vs. 14.5%, *p* = 0.034). Furthermore, a negative correlation of mutation frequency between *IDH1/2* and *DNMT3A* mutations was found among *NPM1* ^mut^ patients (22.2% vs. 49.1%, *p* = 0.010) (Table S3).

**TABLE 1:** Clinical characteristics of the *NPM1* ^mut^ patients.

Characteristic	All cases (*n* = 91)
Age, median (range), years	51 (18–73)
18–39, *n* (%)	24 (26.4)
40–59, *n* (%)	47 (51.6)
60 and older, *n* (%)	20 (22.0)
Gender, male, *n* (%)	49 (53.8)
WBC count, median (range), ×10^9^/L	23.61 (1.09–185.34)
Platelet count, median (range), ×10^9^/L	71.00 (6.00–324.00)
Hemoglobin, median (range), g/L	70.00 (26.79–134.00)
LDH, median (range), U/L	311.00 (76.00–1068.00)
PB blasts, median (range), %	52.50 (0–96.00)
BM blasts, median (range), %	62.50 (22.00–93.50)
Mutations, *n* (%)	
*TET1/2*	52 (57.1)
*IDH1/2*	36 (39.6)
*DNMT3A*	35 (38.5)
MDS‐related genes	35 (38.5)
*FLT3‐TKD*	27 (29.7)
*GATA2*	13 (14.3)
Treatments, *n* (%)	
Transplantation	35 (38.5)
CR/CRi_1_, *n* (%)	61 (67.0)
CR/CRi_2_, *n* (%)	83 (91.2)
MRD‐_1_, *n* (%)	44 (53.0)
MRD‐_2_, *n* (%)	62 (79.5)
Relapse, *n* (%)	11 (12.4)
OS, % (95% CI)	76.2 (55.6, 88.2)
RFS, % (95% CI)	63.8 (42.5, 79.0)

*Note*: MDS‐related genes, which are genes related to myelodysplastic syndrome, include *ASXL1*, *BCOR*, *EZH2*, *RUNX1*, *SF3B1*, *SRSF2*, *STAG2*, *U2AF1*, and *ZRSR2*. Missing values were excluded from the calculation of *p*‐values.

Abbreviations: BM, bone marrow; CR/CRi_1_, percentage of CR/CRi post the first cycle of induction chemotherapy; CR/CRi_2_, percentage of CR/CRi post the 1–2 cycles of induction chemotherapy; LDH, lactate dehydrogenase; MRD‐_1_, percentage of MRD negativity post the 1–2 cycles of induction chemotherapy; MRD‐_2_, percentage of MRD negativity post the first cycle of consolidation chemotherapy; PB, peripheral blood; WBC, white blood cell.

We compared co‐mutation groups, including *TET1/2*, *IDH1/2*, *DNMT3A*, MDS‐related genes, *FLT3‐TKD*, and *GATA2* with their corresponding wild‐type groups. Platelet count was lower in patients with *FLT3‐TKD* ^mut^ than those with *FLT3‐TKD* ^wt^ (49.00 × 10^9^/L vs. 79.00 × 10^9^/L, *p* = 0.020). Patients harboring *DNMT3A* ^mut^ exhibited decreased bone marrow blast percentages (50.00% vs. 64.50%, *p* = 0.017), reduced hemoglobin levels (67.00 g/L vs. 75.50 g/L, *p* = 0.027), and elevated lactate dehydrogenase (403.00 U/L vs. 261.00 U/L, *p* = 0.024) compared to patients with *DNMT3A* ^wt^. Patients carrying *IDH1/2* ^mut^ demonstrated higher bone marrow blast percentages (69.25% vs. 53.00%, *p* = 0.007) as well as peripheral blood blast percentages (69.00% vs. 39.00%, *p* < 0.001) compared to patients with *IDH1/2* ^wt^. Additionally, patients with MDS‐related genes^mut^ displayed lower lactate dehydrogenase levels than those with MDS‐related genes^wt^ (median, 227.00 U/L vs. 352.50 U/L, *p* = 0.016) (Table S3).

When considering *TET1*, *TET2*, *IDH1*, and *IDH2* as independent subtype for analysis, patients with *IDH* ^wt^ had lower percentages of peripheral blood blasts compared to those with *IDH1* ^mut^ (adjusted *p*‐value = 0.032) and *IDH2* ^mut^ (adjusted *p*‐value = 0.024), respectively (Table S4).

### 3.2 Chemotherapeutic response

Among all *NPM1* ^mut^ patients, 67.0% (61/91) patients achieved CR/CRi after the first cycle of induction chemotherapy, while 24.2% (22/91) patients attained CR/CRi following the second cycle of induction chemotherapy. Furthermore, 53.0% (44/83) patients achieved MRD‐negative status after 1–2 cycles of induction chemotherapy, and 79.5% (62/78) after the first cycle of consolidation chemotherapy. Notably, relapse occurred in 12.4% (11/89) patients, with two patients failing to achieve CR/CRi at the end of follow‐up, and a higher relapse rate was observed among patients aged 60 years and older (42.9% vs. 7.6%, *p* = 0.012) (Table 1).

**FIGURE 3:**
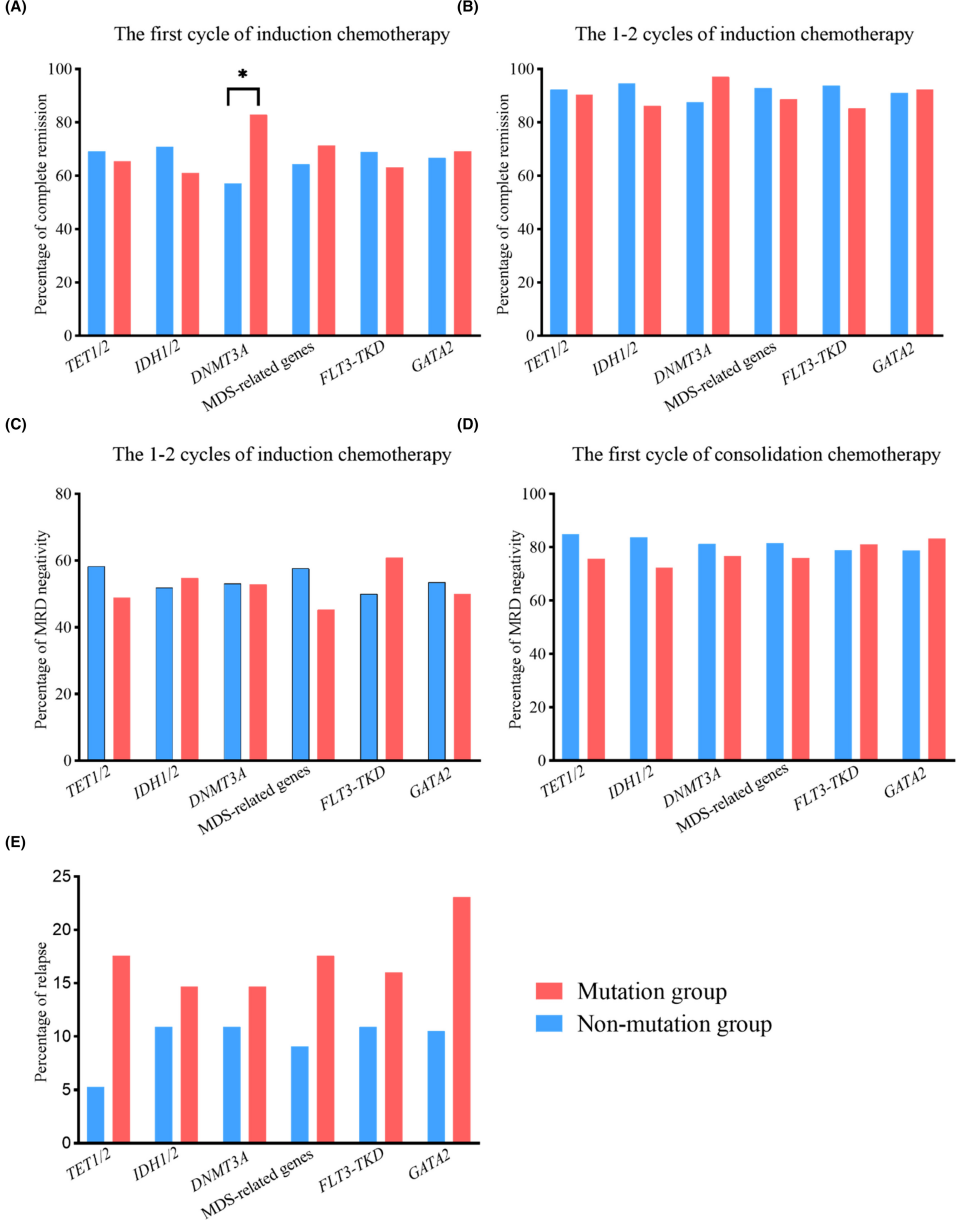
Distribution of CR/CRi, MRD‐negativity and relapse in *NPM1* ^mut^ patients based on co‐mutation status. (A) Patients harboring *DNMT3A* ^mut^ had a significantly higher percentage of CR/CRi after the first cycle of induction chemotherapy. (B) The impact of achieving CR/CRi after 1–2 cycles of induction chemotherapy did not differ by co‐mutation status. (C‐E) Co‐mutations status had no influence on the rate of MRD‐negativity and relapse.

The presence of *DNMT3A* ^mut^ significantly increased the rate of CR/CRi after the first cycle of induction chemotherapy (82.9% vs. 57.1%, *p* = 0.011). However, no significant differences in CR/CRi rates were observed between subgroups without corresponding co‐mutation and those with co‐mutation, including *TET1/2*, *IDH1/2*, MDS‐related genes, *FLT3‐TKD*, and *GATA2* after the first cycle of induction chemotherapy (Figure 3A). The co‐mutation in *TET1/2*, *IDH1/2*, *DNMT3A*, MDS‐related genes, *FLT3‐TKD* and *GATA2* was not found to significantly impact the probability of patients achieving CR/CRi after 1–2 cycles of induction chemotherapy (Figure 3B).

The clearance of MRD did not differ according to mutated status of *TET1/2*, *IDH1/2*, *DNMT3A*, MDS‐related genes, *FLT3‐TKD* and *GATA2* (Figure 3C,D). Additionally, these genetic mutations had no significant influence on the recurrence of *NPM1* ^mut^ patients (Figure 3E).

There was no significant effect on the rates of CR/CRi, MRD negativity or relapse when the analysis of chemotherapeutic response was performed separately for *TET1*, *TET2*, *IDH1*, and *IDH2* (Tables S4 and S5).

### 3.3 Survival

The median overall survival time and relapse‐free survival of *NPM1* ^mut^ patients were not reached (NR). The mean overall survival time for *NPM1* ^mut^ patients was 28.2 months (range 2.3–30 months), and the mean relapse‐free survival was 25.5 months (range 0.7–30 months). Moreover, the rates of OS and RFS at 30‐months were 76.2% (95% CI 55.6%–88.2%) and 63.8% (95% CI 42.5%–79.0%), respectively (Table 1).

**FIGURE 4:**
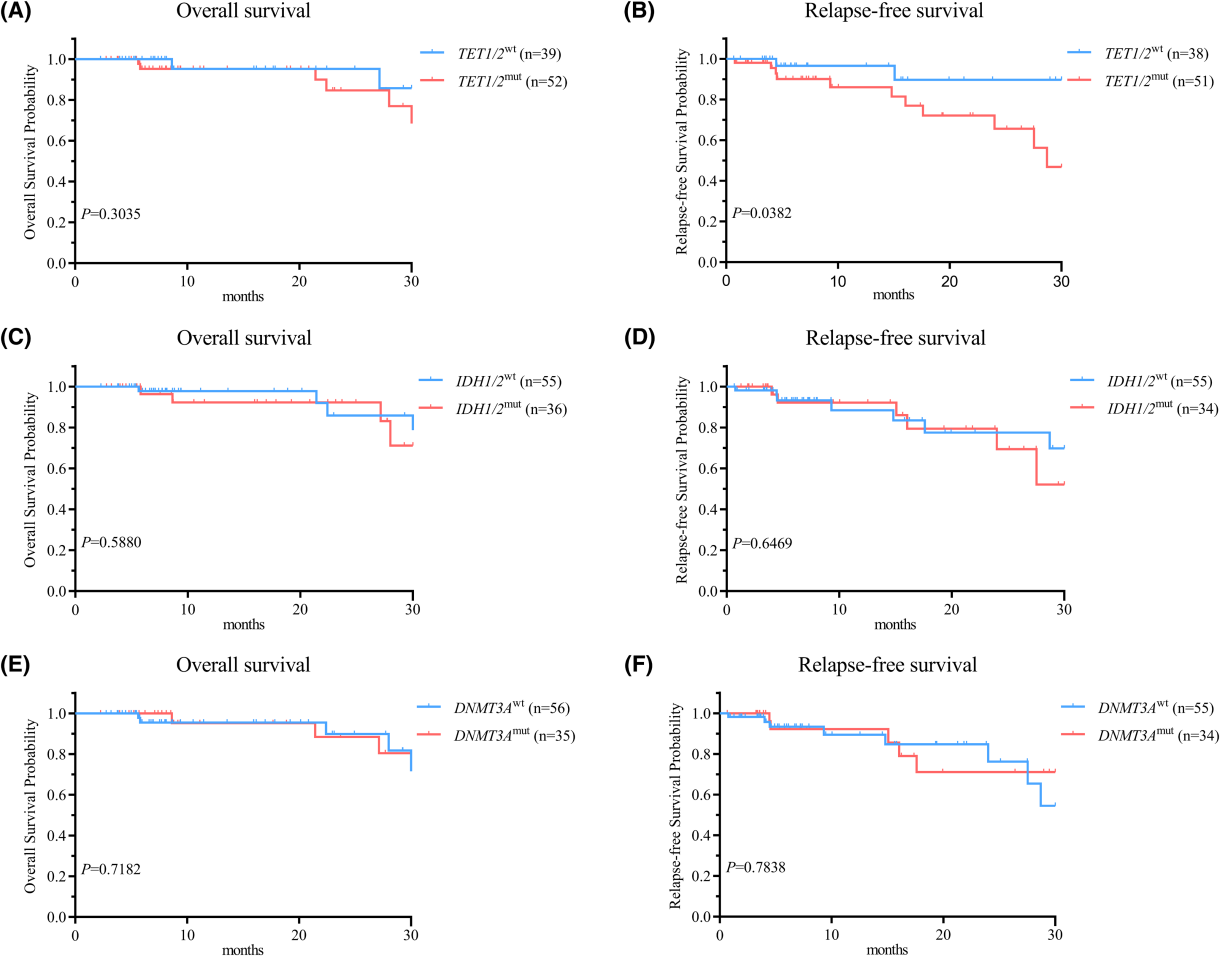
Kaplan–Meier survival analysis of genetic mutation versus corresponding non‐mutaion groups in *NPM1* ^mut^ patients. (A, B) Kaplan–Meier curves comparing *TET1/2* ^mut^ and *TET1/2* ^wt^ showed a significant difference in RFS, but no significant difference was observed in OS. (C–F) *IDH1/2* ^mut^ and *DNMT3A* ^mut^ had no significant impact on OS and RFS.

**FIGURE 5:**
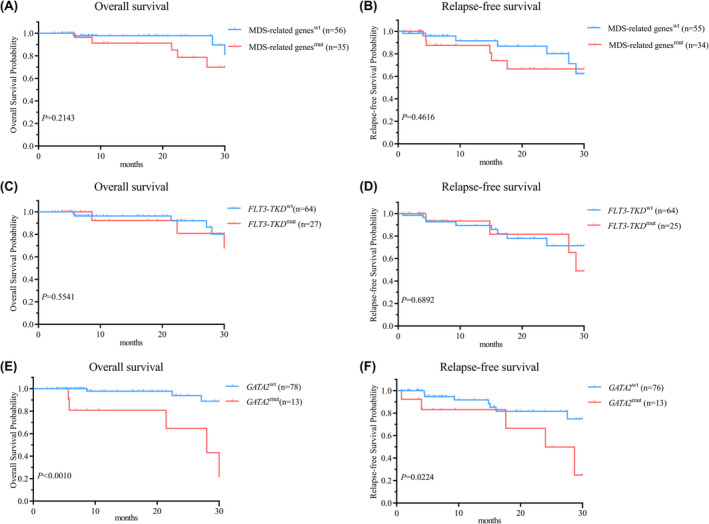
Kaplan–Meier survival analysis of genetic mutation versus corresponding non‐mutaion groups in *NPM1* ^mut^ patients. (A–D) MDS‐related genes^mut^ and *FLT3‐TKD* ^mut^ had no significant influence on OS and RFS. (E, F) Kaplan–Meier curves comparing *GATA2* ^mut^ and *GATA2* ^wt^ showed a significant difference in OS and RFS.

Univariate survival analysis indicated that patients with *TET1/2* ^mut^ had worse RFS (median, 28.7 vs. not NR months; *p* = 0.0382) but not OS (median, NR vs. NR; *p* = 0.3035) than those with *TET1/2* ^wt^ (Figure 4A,B). *GATA2* ^mut^ subtype was associated with inferior OS (median, 28 vs. NR months; *p* < 0.0010) and RFS (median, 24 vs. NR months; *p* = 0.0224) compared to the *GATA2* ^wt^ subtype (Figure 5E,F). Co‐mutation in *IDH1/2*, *DNMT3A*, *FLT3‐TKD* and MDS‐related genes did not exert a significant effect on OS and RFS (Figures 4C–F and 5A–D). Equivalent findings were observed when the analysis was restricted to patients with normal karyotype or individuals aged 60 years or younger (Table S6).

Additionally, the survival effect of *IDH1* and *IDH2* was analyzed separately and did not show statistical significance. Although patients with *TET1* ^mut^/*TET2* ^mut^ had a shorter RFS than those with *TET* ^wt^ (median, NR vs. NR; *p* = 0.0272), the sample size of *TET1* ^mut^/*TET2* ^mut^ was small and only provided reference value (Figure S1).

**FIGURE 6:**
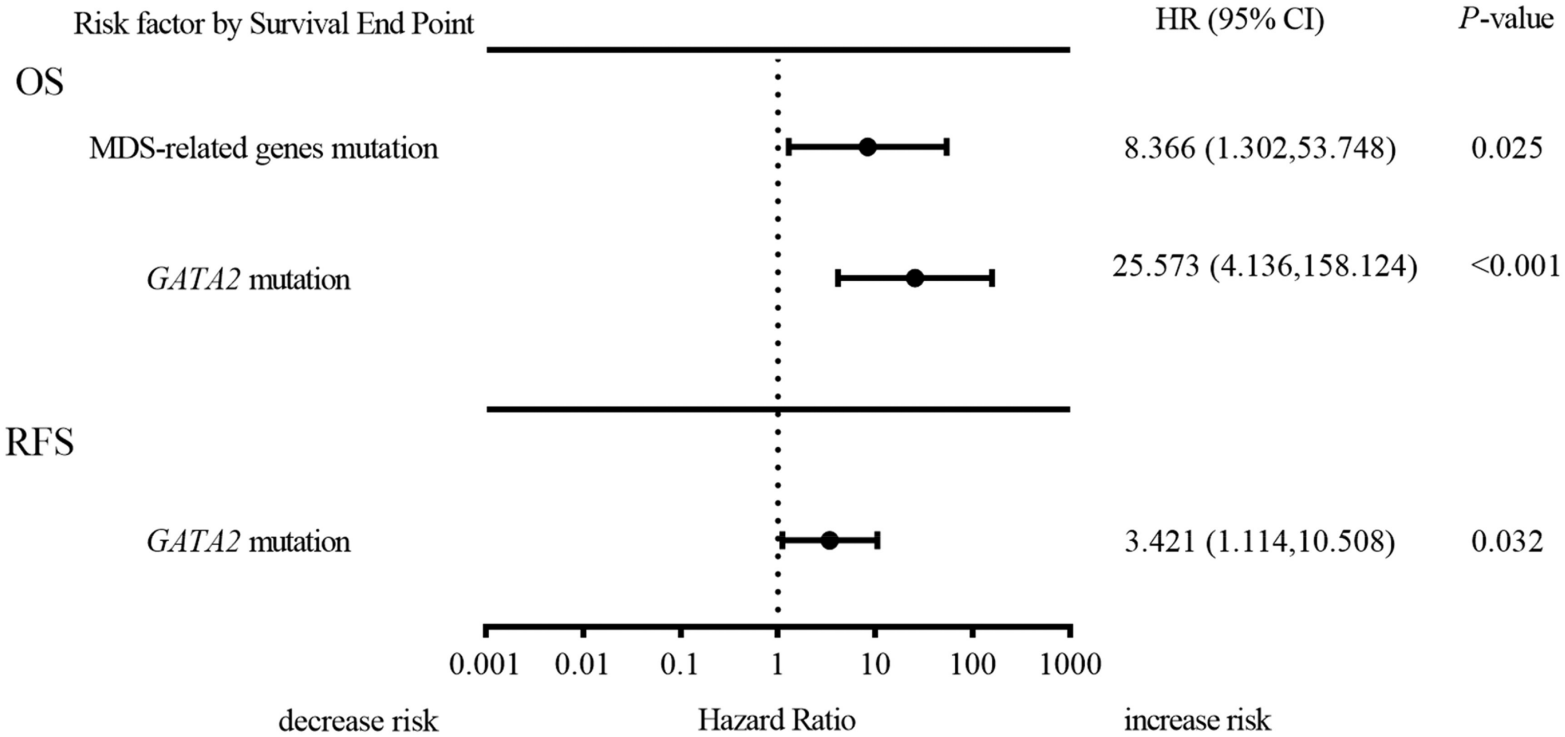
Multivariable Cox regression analysis of OS and RFS in *NPM1* ^mut^ patients (*n* = 91). Sex, age, WBC count, platelet count, hemoglobin level, LDH level, BM blasts percentages, PB blasts percentages, abnormal karyotype, and co‐mutation status (*TET1/2*, *IDH1/2*, *DNMT3A*, MDS‐related genes, *FLT3‐TKD*, and *GATA2* mutations) were analyzed by univariate Cox regression analysis. Variables with a *p*‐value of less than 0.5 in univariate Cox‐regression analysis were selected for inclusion in the multivariable Cox regression analysis. The optimal combination of covariates for multivariable Cox regression was determined via a stepwise selection procedure.

Cox regression analysis was conducted to assess the impact of sex, age, white blood count, platelet count, hemoglobin level, lactate dehydrogenase level, bone marrow blasts percentages, peripheral blasts percentages, abnormal karyotype, and co‐mutation status on OS and RFS (Tables S7 and S8). The results revealed that patients with *GATA2* ^mut^ had a significantly higher risk of death with a hazard ratio (HR) of 25.573 (95% CI 4.136–158.124), while those with MDS‐related genes^mut^ had an increased risk of death with an HR of 8.366 (95% CI 1.302–53.748). Patients with *GATA2* ^mut^ had a 3.421‐fold increased hazard of recurrence (HR, 3.421; 95% CI 1.114–10.508) (Figure 6).

## 4 DISCUSSION

The existing research findings suggest that *NPM1* ^mut^ AML is a subtype characterized by genetic and clinical heterogeneity, rather than being a homogeneous entity. The investigation of multiple concomitant mutations has significant implications on formulating an optimal therapeutic strategy. This study implied that mutations in *TET1/2*, *GATA2* and MDS‐related genes should be considered when assessing chemotherapeutic outcome in *NPM1* ^mut^ AML patients.

The favorable prognosis of *NPM1* ^mut^/*FLT3‐ITD* ^wt^ patients within our study corresponds to that presented in recent studies.^14^, ^17^, ^18^ Compared to two previous cohorts with similar median age, the frequencies of *TET1/2*, *IDH1/2*, *FLT3‐TKD*, *GATA2*, and MDS‐related genes mutations in our study were moderately higher, whereas the frequency of *DNMT3A* mutation was slightly lower.^4^, ^19^, ^20^ The slight difference may be attributed to the exclusion of *FLT3‐ITD* ^mut^ patients. The limitation of our cohort to patients with intermediate‐risk karyotypes may constitute additional factor. Although a favorable prognosis was a feature common to the majority of patients with *NPM1* ^mut^ AML, poor clinical outcomes were observed in some patients, which did seem to be related to the presence of these co‐mutations. We noted that the effect of *TET1/2*, *GATA2*, and MDS‐related genes mutations on chemotherapeutic outcome was prominent. Notably, we observed a significant difference in RFS between patients with *TET1/2* ^mut^ and *TET1/2* ^wt^, but not in OS. The fact that a proportion of relapsed patients were still alive at the end of follow‐up probably accounts for this. The relatively limited duration of the follow‐up may potentially obscure the effects of *TET1/2* ^mut^ on OS. Remarkably, contrary to the findings of multivariable Cox regression analysis, the results of univariate analysis did not indicate survival difference in MDS‐related genes, even within subgroups characterized by normal karyotype or individuals below 60 years old. The impact of MDS‐related genes on survival is revealed when controlling for covariates. It cannot be ruled out that there may exist potential confounding factors, which were not accounted for in this study. Furthermore, considering the unique composition of clustered multiple genes associated with MDS, it is imperative to investigate whether patients within this group can be defined as a homogeneous cohort.

There have been conflicting conclusions regarding whether these genes are independently associated with survival in *NPM1* ^mut^ AML. The results from several groups demonstrated that *TET1/2*, *IDH1/2* and MDS‐related genes were linked to unfavorable prognosis in *NPM1* ^mut^ AML.^21^, ^22^, ^23^, ^24^, ^25^, ^26^ Several research studies have uncovered synergistic positive effects on the prognosis of *NPM1* ^mut^ AML when *FLT3‐TKD*, *IDH1/2* co‐occur.^27^, ^28^, ^29^ In contrast, other studies did not observe significant associations between the co‐occurrence of *TET1/2*, *IDH1/2*, *DNMT3A*, MDS‐related genes and prognosis in *NPM1* ^mut^ AML.^30^, ^31^, ^32^, ^33^, ^34^, ^35^ The adverse survival events in *NPM1* ^mut^ AML group may not be solely explained by the double‐mutant type. Previous studies have described the triple‐mutant type that is correlated with an inferior prognosis in *NPM1* ^mut^ AML.^36^, ^37^, ^38^ It emphasizes the criticality of exploring wider genomic context to constitute a more robust strategy for prognostic stratification.

The pathogenic dysfunction of *GATA2* gene is increasingly gaining significance in clinical management of leukemia. *MDS1*‐*EVI1* complex (MECOM) rearrangements, which involve the *GATA2* gene, have been identified as an adverse risk factor in AML based on the 2022 European LeukemiaNet risk stratification.^12^, ^39^, ^40^, ^41^ The *GATA2* mutation was classified as a clonal hematopoiesis of oncogenic potential (CHOP‐like) mutation, which predicts an adverse prognosis when it persists or is acquired post‐remission.^42^ Some research findings confirmed that overexpression of *GATA2* predicted an adverse prognosis in AML patients.^43^, ^44^, ^45^ However, given the infrequent occurrence of *GATA2* mutation^4^, ^19^ there is a lack of studies investigating its survival effect in the *NPM1* ^mut^ subgroup. Our data uncover a previously unrecognized role of *GATA2* mutation as a poor prognostic marker in *NPM1* ^mut^ AML. The relevant studies have reported that mutant NPM1 increased the expression of *GATA2* through bounding to the −77 kb enhancer region of *GATA2*.^11^ A Speculative interpretation is that mutant NPM1 may disturb activation of *GATA2*‐mediated transcription in *NPM1* ^mut^/*GATA2* ^mut^ AML.

In contrast to our findings, a recent study indicated that patients with *DNMT3A* ^mut^ did not exert a significant impact on CR rate.^33^ Studies have illustrated that lower bone marrow blast percentages confer an improved chemotherapeutic outcome.^12^, ^46^, ^47^ This may explain why cases with *DNMT3A* ^mut^ showed a higher rate of CR/CRi in our cohort. Monitoring the MRD level has an important value in prognostic evaluation and therapeutic stratification. FCM was used to monitor MRD in this study. However, the specificity of LAIPs in *NPM1* ^mut^ AML patients is not absolute, particularly in cases presenting significant monocytic differentiation at diagnosis or exhibiting a predominance of monocytic residual diseases.^48^, ^49^, ^50^ Moreover, the presence of persistent clonal hematopoiesis‐associated mutations may lead to spurious detection of MRD by FCM following eradication of *NPM1* mutation.^51^, ^52^, ^53^ Therefore, our data may not fully represent the impact of concomitant mutations on MRD. The combination of FCM and molecular detection exhibits enhanced efficiency in evaluating MRD levels.

Although this study has significant implications for precision treatment, the conclusion drawn from the retrospective study should be interpreted with caution. Further research, including multicenter studies with larger samples and prospective studies, is needed to validate these observations. Unfortunately, the availability of both germline and remission samples was limited in this study. Therefore, we could not determine whether these mutations were somatic or germline.

In summary, our retrospective study confirmed that *TET1/2*, *GATA2* and MDS‐related genes mutations were related to poor therapeutic outcomes in patients with *NPM1* ^mut^ AML. These findings provided valuable insights for refining risk stratification, improving prognosis assessment, and optimizing treatment strategies in individuals with *NPM1* ^mut^ AML.

## AUTHOR CONTRIBUTIONS

**Quan Wu:** Conceptualization (equal); data curation (equal); formal analysis (equal); investigation (equal); methodology (equal); visualization (equal); writing – original draft (equal). **Yujiao Zhang:** Investigation (equal); writing – review and editing (equal). **Baoyi Yuan:** Data curation (equal); investigation (equal). **Yun Huang:** Methodology (equal); software (equal). **Ling Jiang:** Methodology (equal); software (equal). **Fang Liu:** Methodology (equal); software (equal). **Ping Yan:** Methodology (equal); software (equal). **Jiaying Cheng:** Methodology (equal); software (equal). **Zhiquan Long:** Software (equal). **Xuejie Jiang:** Conceptualization (equal); funding acquisition (equal); project administration (equal); resources (equal); supervision (equal); writing – review and editing (equal).

## FUNDING INFORMATION

This study was supported by the National Natural Science Foundation of China (82170165); the Natural Science Foundation of Guangdong Province (2023A1515012401); and the Natural Science Foundation of Guangdong Province (2021A1515011437).

## CONFLICT OF INTEREST STATEMENT

The authors have no competing financial interests. None of the authors are members of the Cancer Medicine editorial board or current editors.

## ETHICS STATEMENT

This study was conducted in compliance with the 1964 Helsinki Declaration and the ethical standards established by institutional and/or national research committees. Due to the retrospective nature of this study, the informed consent requirement was waived, and it was determined to be exempt from review by the Ethics Committee.

## Supporting information


Figure S1.

Table S1.

Table S2.

Table S3.

Table S4.

Table S5.

Table S6.

Table S7.

Table S8.

Table S9.

## Data Availability

The data supporting the findings of this study can be provided upon reasonable request, taking into account privacy or ethical constraints.
