# Supplementary material for: Influence of genetic co‐mutation on chemotherapeutic outcome in NPM1‐mutated and FLT3‐ITD wild‐type AML patients
Source: Cancer Med. 2024 Aug 9;13(15):e70102. doi: 10.1002/cam4.70102 (PMC11316012; doi:10.1002/cam4.70102)
Supplement: Supplementary file 1 — Figure S1. [file CAM4-13-e70102-s006.docx]

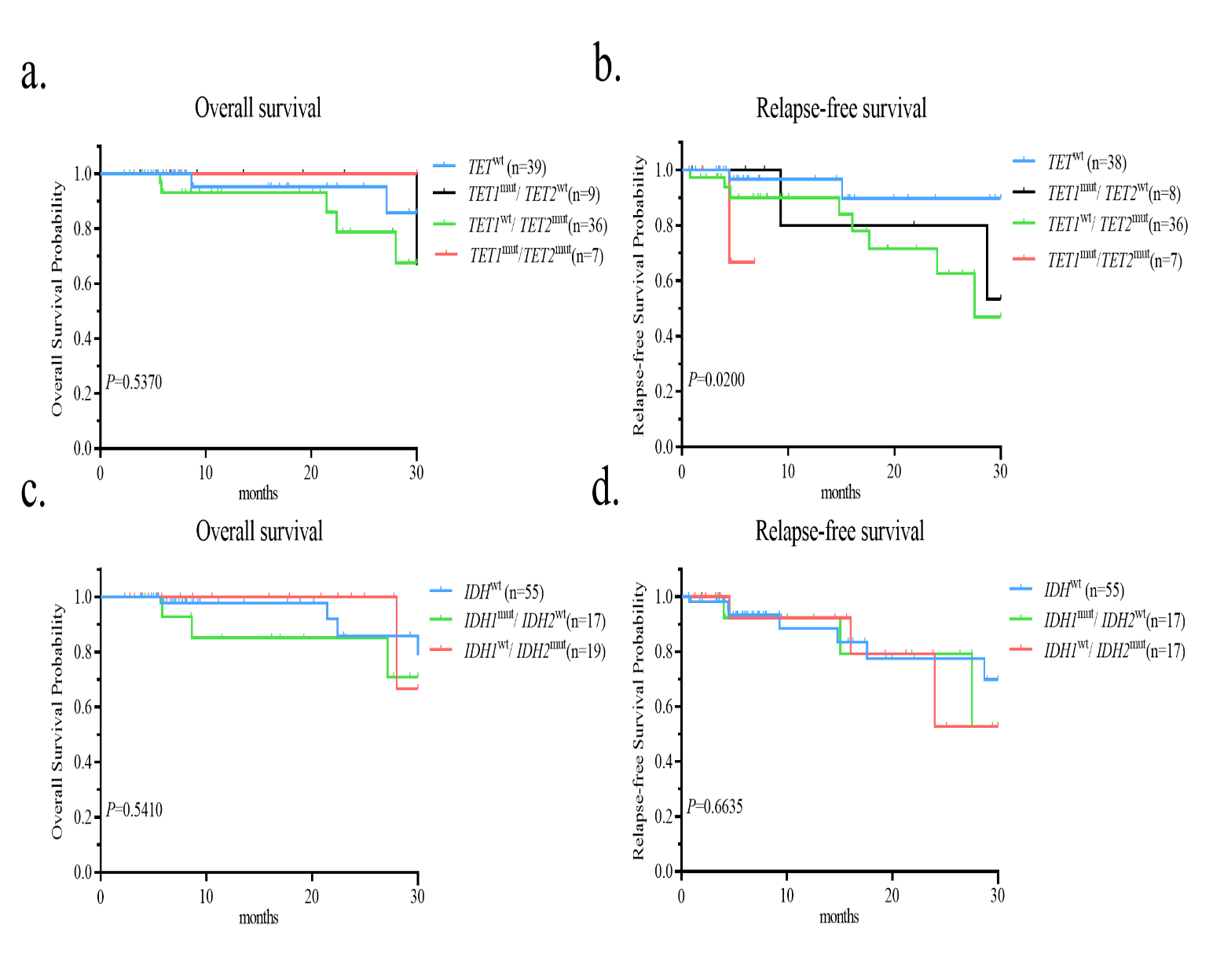
Figure S1. Kaplan-Meier curves according to *TET* and *IDH* status. (a) Kaplan-Meier curves comparing *TET*^wt^, *TET1*^mut^/*TET2*^wt^, *TET1*^wt^/*TET2*^mut^ and *TET1*^mut^/*TET2*^mut^ showed no significant difference in OS. (b) Patients with *TET1*^mut^/*TET2*^mut^ had a shorter RFS than those with *TET*^wt^. (c-d) Kaplan-Meier curves comparing *IDH*^wt^, *IDH1*^mut^/*IDH2*^wt^ and *IDH1*^wt^/*IDH2*^mut^ showed no significant difference in OS and RFS.
