# Supplementary material for: Influence of genetic co‐mutation on chemotherapeutic outcome in NPM1‐mutated and FLT3‐ITD wild‐type AML patients
Source: Cancer Med. 2024 Aug 9;13(15):e70102. doi: 10.1002/cam4.70102 (PMC11316012; doi:10.1002/cam4.70102)
Supplement: Supplementary file 2 — Table S1. [file CAM4-13-e70102-s009.docx]

| ASXL1 | CBL | DNMT3A | CALR | ETV6 |
| --- | --- | --- | --- | --- |
| EZH2 | IDH1 | IDH2 | MPL | JAK2 |
| NRAS | SH2B3 | KRAS | RUNX1 | SETBP1 |
| CSF3R | SF3B1 | SRSF2 | TET2 | BCOR |
| TP53 | U2AF1 | ZRSR2 | BCORL1 | PIGA |
| CEBPA | FLT3 | KIT | NPM1 | GATA2 |
| MLL | PDGFRA | PHF6 | WT1 | STAT3 |
| PPMID | NF1 | STAG2 |  |  |

Supplement table 1. The NGS-panel used for the detection of genetic mutations in this study is listed below.
