# Supplementary material for: Influence of genetic co‐mutation on chemotherapeutic outcome in NPM1‐mutated and FLT3‐ITD wild‐type AML patients
Source: Cancer Med. 2024 Aug 9;13(15):e70102. doi: 10.1002/cam4.70102 (PMC11316012; doi:10.1002/cam4.70102)
Supplement: Supplementary file 3 — Table S2. [file CAM4-13-e70102-s010.docx]

Table S2. Cases with abnormal karyotypes.

| Case | Karyotype |
| --- | --- |
| 1  2  3  4  5  6  7  8  9  10 | 45,X,-X/46,XX  45,X,-Y/46,XY  46,XX+mar/46,XX  46,XY,i(7)(q10)  46,XY,inv(11)(q13q24)/46,XY  46~47,XX,+4,del(9)(q13q22)  46,XY,inv(Y)(p11q11)  47,XX,+8/46,XX  46,XX,del(21)(q21q22)/46,XX  47,XY,+10 |
