# Supplementary material for: Influence of genetic co‐mutation on chemotherapeutic outcome in NPM1‐mutated and FLT3‐ITD wild‐type AML patients
Source: Cancer Med. 2024 Aug 9;13(15):e70102. doi: 10.1002/cam4.70102 (PMC11316012; doi:10.1002/cam4.70102)
Supplement: Supplementary file 4 — Table S3. [file CAM4-13-e70102-s007.docx]

Table S3. Characteristics of patients according to co-mutation status.

| Characteristic | *TET1/2*^wt^  (n=39) | *TET1/2*^mut^  (n=52) | *P* | *IDH1/2*^wt^  (n=55) | *IDH1/2*^mut^  (n=36) | *P* | *DNMT3A*^wt^  (n=56) | *DNMT3A*^mut^  (n=35) | *P* |
| --- | --- | --- | --- | --- | --- | --- | --- | --- | --- |
| Age, median, y  60 and older, n (%) | 51.0  23.1 | 51.0  21.2 | 0.844  0.826 | 51.0  14.5 | 53.5  33.3 | 0.330  **0.034** | 47.5  17.9 | 52.0  28.6 | 0.110  0.230 |
| Sex, male, n (%) | 48.7 | 57.7 | 0.395 | 60.0 | 44.4 | 0.146 | 58.9 | 45.7 | 0.219 |
| WBC count, median, ×10^9^/L | 28.39 | 22.29 | 0.501 | 21.99 | 28.00 | 0.644 | 22.13 | 28.74 | 0.153 |
| Platelet count, median, ×10^9^/L | 74.00 | 67.50 | 0.691 | 71.00 | 71.50 | 0.884 | 74.50 | 67.00 | 0.446 |
| Hemoglobin, median, g/L | 71.00 | 69.00 | 0.904 | 68.00 | 70.50 | 0.814 | 75.50 | 67.00 | **0.027** |
| LDH, median, U/L | 320.00 | 276.00 | 0.661 | 323.00 | 270.50 | 0.789 | 261.00 | 403.00 | **0.024** |
| PB blasts, median, % | 56.50 | 49.50 | 0.844 | 39.00 | 69.00 | **<0.001** | 63.00 | 39.00 | 0.062 |
| BM blasts, median, % | 60.00 | 63.00 | 1.000 | 53.00 | 69.25 | **0.007** | 64.50 | 50.00 | **0.017** |
| Mutations, n (%)  *TET1/2*  *IDH1/2*  *DNMT3A*  MDS-related genes  *FLT3-TKD*  *GATA2* | -  41.0  46.2  41.0  33.3  7.7 | -  38.5  32.7  36.5  26.9  19.2 | -  0.804  0.191  0.663  0.508  0.120 | 58.2  -  49.1  36.4  29.1  9.1 | 55.6  -  22.2  41.7  30.6  22.2 | 0.804  -  **0.010**  0.611  0.881  0.080 | 62.5  50.0  -  42.9  28.6  19.6 | 48.6  22.9  **-**  31.4  31.4  5.7 | 0.191  **0.010**  -  0.276  0.772  0.065 |
| treatments, n (%)  transplantation | 38.5 | 38.5 | 1.000 | 43.6 | 30.6 | 0.210 | 42.9 | 31.4 | 0.276 |
| CR/CRi_1_, n (%)  CR/CRi_2_, n (%)  MRD-_1_, n (%)  MRD-_2_, n (%)  Relapse, n (%) | 69.2  92.3  58.3  84.8  5.3 | 65.4  90.4  48.9  75.6  17.6 | 0.699  1.000  0.395  0.315  0.107 | 70.9  94.5  51.9  83.7  10.9 | 61.1  86.1  54.8  72.4  14.7 | 0.331  0.256  0.797  0.234  0.742 | 57.1  87.5  53.1  81.3  10.9 | 82.9  97.1  52.9  76.7  14.7 | **0.011**  0.146  0.991  0.626  0.742 |

Table S3 (continue). Characteristics of patients according to co-mutation status.

| Characteristic | MDS-related genes^wt^  (n=56) | MDS-related genes^mut^  (n=35) | *P* | *FLT3-TKD*^wt^  (n=64) | *FLT3-TKD*^mut^  (n=27) | *P* | *GATA2*^wt^  (n=78) | *GATA2*^mut^  (n=13) | *P* |
| --- | --- | --- | --- | --- | --- | --- | --- | --- | --- |
| Age, median, y  60 and older, n (%) | 49.0  16.1 | 53.0  31.4 | 0.134  0.085 | 51.0  21.9 | 51.0  22.2 | 0.741  0.971 | 51.0  21.8 | 43.0  23.1 | 0.638  1.000 |
| Sex, male, n (%) | 57.1 | 48.6 | 0.425 | 56.3 | 48.1 | 0.479 | 52.6 | 61.5 | 0.548 |
| WBC count, median, ×10^9^/L | 22.96 | 25.56 | 0.525 | 20.04 | 39.32 | 0.051 | 22.29 | 25.56 | 0.777 |
| Platelet count, median, ×10^9^/L | 63.50 | 82.00 | 0.060 | 79.00 | 49.00 | **0.020** | 69.50 | 78.00 | 0.725 |
| Hemoglobin, median, g/L | 71.00 | 69.00 | 0.750 | 69.50 | 71.00 | 0.913 | 69.50 | 73.00 | 0.892 |
| LDH, median, U/L | 352.50 | 227.00 | **0.016** | 320.00 | 300.00 | 0.543 | 326.00 | 274.00 | 0.471 |
| PB blasts, median, % | 58.50 | 43.00 | 0.120 | 45.00 | 62.00 | 0.441 | 48.00 | 64.00 | 0.644 |
| BM blasts, median, % | 62.75 | 62.00 | 0.568 | 54.75 | 65.00 | 0.056 | 59.00 | 67.00 | 0.170 |
| Mutations, n (%)  *TET1/2*  *IDH1/2*  *DNMT3A*  MDS-related genes  *FLT3-TKD*  *GATA2* | 58.9  37.5  42.9  -  33.9  16.1 | 54.3  42.9  31.4  -  22.9  11.4 | 0.663  0.611  0.276  -  0.261  0.538 | 59.4  39.1  37.5  42.2  -  14.1 | 51.9  40.7  40.7  29.6  -  14.8 | 0.508  0.881  0.772  0.261  -  1.000 | 53.8  35.9  42.3  39.7  29.5  - | 76.9  61.5  15.4  30.8  30.8  - | 0.120  0.080  0.065  0.538  1.000  - |
| treatments, n (%)  transplantation | 41.1 | 34.3 | 0.517 | 40.6 | 33.3 | 0.514 | 41.0 | 23.1 | 0.218 |
| CR/CRi_1_, n (%)  CR/CRi_2_, n (%)  MRD-_1_, n (%)  MRD-_2_, n (%)  Relapse, n (%) | 64.3  92.9  57.7  81.6  9.1 | 71.4  88.6  45.2  75.9  17.6 | 0.481  0.479  0.269  0.542  0.322 | 68.8  93.8  50.0  78.9  10.9 | 63.0  85.2  60.9  81.0  16.0 | 0.592  0.321  0.375  1.000  0.495 | 66.7  91.0  53.5  78.8  10.5 | 69.2  92.3  50.0  83.3  23.1 | 1.000  1.000  0.821  1.000  0.199 |

CR/CRi_1_, percentage of CR/CRi post the first cycle of induction chemotherapy; CR/CRi_2,_ percentage of CR/CRi post the 1-2 cycles of induction chemotherapy; MRD-_1_, percentage of MRD negativity post the 1-2 cycles of induction chemotherapy; MRD-_2_, percentage of MRD negativity post the first cycle of consolidation chemotherapy. Missing values were excluded from the calculation of *P*-values. The results considered statistically significant are presented in bold.
