# Supplementary material for: Influence of genetic co‐mutation on chemotherapeutic outcome in NPM1‐mutated and FLT3‐ITD wild‐type AML patients
Source: Cancer Med. 2024 Aug 9;13(15):e70102. doi: 10.1002/cam4.70102 (PMC11316012; doi:10.1002/cam4.70102)
Supplement: Supplementary file 5 — Table S4. [file CAM4-13-e70102-s001.docx]

Table S4. Patient characteristics according to *IDH* mutation status.

| Characteristic | *IDH*^wt^ | *IDH1*^mut^/*IDH2*^wt^ | *IDH1*^wt^*/IDH2*^mut^ | *P-*Value |
| --- | --- | --- | --- | --- |
| Number, n | 55 | 17 | 19 | - |
| Age, median (range), y | 51 (22-69) | 57 (18-70) | 49 (22-73) | 0.357 |
| Sex, male, n (%) | 33 (60.0) | 9 (52.9) | 7 (36.8) | 0.217 |
| WBC count, median (range), ×10^9^/L | 21.99 (1.09-162.10) | 29.58 (1.39-185.34) | 26.42 (1.65-112.64) | 0.868 |
| Platelet count, median (range), ×10^9^/L | 71.00 (10.30-324.00) | 69.00 (6.00-288.00) | 74.00 (6.00-318.00) | 0.978 |
| Hemoglobin, median (range), g/L | 68.00 (26.79-120.00) | 75.00 (41.00-112.00) | 69.00 (45.00-134.00) | 0.717 |
| LDH, median (range), U/L | 323.00 (76.00-846.00) | 337.00 (130.00-1068.00) | 247.50 (173.00-580.00) | 0.398 |
| PB blasts, median (range), % | 39.00 (0-96.00) | 61.50 (27.00-94.00) | 73.00 (14.00-93.00) | 0.004^a^ |
| BM blasts, median (range), % | 53.00 (22.00-92.00) | 63.50 (40.00-93.50) | 74.00 (32.00-91.50) | 0.0280^b^ |
| Treatments, n (%)  Transplantation | 24 (43.6) | 4 (23.5) | 7 (36.8) | 0.325 |
| CR/CRi_1_, n (%)  CR/CRi_2_, n (%)  MRD-_1_, n (%)  MRD-_2_, n (%)  Relapse, n (%) | 39 (70.9)  52 (94.5)  27 (51.9)  41 (83.7)  6 (10.9) | 9 (52.9)  14 (82.4)  7 (50.0)  8 (61.5)  3 (20.0) | 13 (68.4)  17 (89.5)  10 (58.8)  13 (81.3)  2 (10.5) | 0.424  0.183  0.903  0.222  0.587 |

(a) Patients with *IDH*^wt^ had lower percentages of peripheral blood blasts compared to those with *IDH1*^mut^ (adjusted *p*-value=0.032) and *IDH2*^mut^ (adjustive *p*-value=0.024), respectively. (b) The pairwise comparisons among the three variables did not yield any statistically significant results. CR/CRi_1_, percentage of CR/CRi post the first cycle of induction chemotherapy; CR/CRi_2,_ percentage of CR/CRi post the 1-2 cycles of induction chemotherapy; MRD-_1_, percentage of MRD negativity post the 1-2 cycles of induction chemotherapy; MRD-_2_, percentage of MRD negativity post the first cycle of consolidation chemotherapy. Missing values were excluded from the calculation of *P*-values.
