# Supplementary material for: Influence of genetic co‐mutation on chemotherapeutic outcome in NPM1‐mutated and FLT3‐ITD wild‐type AML patients
Source: Cancer Med. 2024 Aug 9;13(15):e70102. doi: 10.1002/cam4.70102 (PMC11316012; doi:10.1002/cam4.70102)
Supplement: Supplementary file 6 — Table S5. [file CAM4-13-e70102-s004.docx]

Table S5. Patient characteristics according to *TET* mutation status.

| Characteristic | *TET*^wt^ | *TET1*^mut^*/TET2*^mut^ | *TET1*^mut^/*TET2*^wt^ | *TET1*^wt^*/TET2*^mut^ | *P-*Value |
| --- | --- | --- | --- | --- | --- |
| Number, n | 39 | 7 | 9 | 36 | - |
| Age, median (range), y | 51 (18-70) | 53 (26-69) | 52 (22-73) | 41 (29-68) | 0.480 |
| Sex, male, n (%) | 19 (48.7) | 4 (57.1) | 5 (55.6) | 21 (58.3) | 0.862 |
| WBC count, median (range), ×10^9^/L | 28.39 (1.40-185.34) | 51.80 (1.94-55.37) | 22.27 (1.69-147.31) | 21.24 (1.09-115.04) | 0.709 |
| Platelet count, median (range), ×10^9^/L | 74.00 (6.00-288.00) | 63.00 (23.00-119.00) | 51.00 (14.00-324.00) | 76.50 (13.00-318.00) | 0.777 |
| Hemoglobin, median (range), g/L | 71.00 (26.79-134.00) | 80.00 (58.00-100.00) | 66.00 (41.00-104.00) | 68.50 (45.00-120.00) | 0.554 |
| LDH, median (range), U/L | 320.00 (76.00-1068.00) | 273.50 (110.00-722.00) | 285.50 (130.00-624.00) | 276.00 (80.00-846.00) | 0.957 |
| PB blasts, median (range), % | 56.50 (0-94.00) | 48.00 (16.00-80.00) | 35.00 (10.00-96.00) | 62.00 (1.00-88.00) | 0.952 |
| BM blasts, median (range), % | 60.00 (25.00-91.50) | 58.50 (23.50-92.00) | 64.50 (24.00-93.50) | 62.50 (22.00-90.00) | 0.977 |
| Treatments, n (%)  Transplantation | 15 (38.5) | 6 (85.7) | 3 (33.3) | 11 (30.6) | 0.054 |
| CR/CRi_1_, n (%)  CR/CRi_2_, n (%)  MRD-_1_, n (%)  MRD-_2_, n (%)  Relapse, n (%) | 27 (69.2)  36 (92.3)  21 (58.3)  28 (84.8)  2 (5.3) | 2 (28.6)  5 (71.4)  3 (60.0)  4 (80.0)  1 (14.3) | 5 (55.6)  7 (77.8)  4 (57.1)  5 (83.3)  2 (25.0) | 27 (75.0)  35 (97.2)  16 (45.7)  25 (73.5)  6 (16.7) | 0.096  0.053  0.742  0.766  0.194 |

CR/CRi_1_, percentage of CR/CRi post the first cycle of induction chemotherapy; CR/CRi_2,_ percentage of CR/CRi post the 1-2 cycles of induction chemotherapy; MRD-_1_, percentage of MRD negativity post the 1-2 cycles of induction chemotherapy; MRD-_2_, percentage of MRD negativity post the first cycle of consolidation chemotherapy. Missing values were excluded from the calculation of *P*-values.
