# Supplementary material for: Influence of genetic co‐mutation on chemotherapeutic outcome in NPM1‐mutated and FLT3‐ITD wild‐type AML patients
Source: Cancer Med. 2024 Aug 9;13(15):e70102. doi: 10.1002/cam4.70102 (PMC11316012; doi:10.1002/cam4.70102)
Supplement: Supplementary file 7 — Table S6. [file CAM4-13-e70102-s003.docx]

Table S6. Kaplan-Meier survival analysis was conducted for patients with normal karyotypes or who were below 60 years of age.

| Variable | Number  (n) | |  | Median overall survival time  (months) | | |  | Median relapse-free survival  (months) | | |
| --- | --- | --- | --- | --- | --- | --- | --- | --- | --- | --- |
|  | Wild-type | Mutant |  | Wild-type | Mutant | *P* |  | Wild-type | Mutant | *P* |
| Patients with normal karyotypes | | | | | | | | | | |
| *TET1/2*  *IDH1/2*  *DNMT3A*  MDS-related genes  *FLT3-TKD*  *GATA2* | 34  49  49  49  60  70 | 47  32  32  32  21  11 |  | NR  NR  NR  NR  NR  NR | NR  NR  NR  NR  NR  28.0 | 0.559  0.322  0.830  0.107  0.787  <0.001 |  | NR  NR  NR  NR  NR  NR | NR  27.5  NR  NR  NR  24.0 | 0.031  0.232  0.921  0.586  0.597  0.034 |
| Patients aged <60 years | | | | | | | | | | |
| *TET1/2*  *IDH1/2*  *DNMT3A*  MDS-related genes  *FLT3-TKD*  *GATA2* | 30  47  46  47  50  61 | 41  24  25  24  21  10 |  | -  NR  -  NR  NR  NR | -  28.0  -  NR  NR  28.0 | -  0.450  -  0.503  0.694  <0.001 |  | -  NR  NR  NR  NR  NR | -  NR  NR  NR  NR  28.7 | -  0.604  0.301  0.780  0.879  0.005 |

NR, not reached. Certain subgroups were not subjected to survival analysis due to the censorship of all patients.
