# Supplementary material for: Influence of genetic co‐mutation on chemotherapeutic outcome in NPM1‐mutated and FLT3‐ITD wild‐type AML patients
Source: Cancer Med. 2024 Aug 9;13(15):e70102. doi: 10.1002/cam4.70102 (PMC11316012; doi:10.1002/cam4.70102)
Supplement: Supplementary file 8 — Table S7. [file CAM4-13-e70102-s002.docx]

| Variable | Univariate analysis | |  | Multivariate analysis | |
| --- | --- | --- | --- | --- | --- |
|  | HR (95%CI) | *P* |  | HR (95%CI) | *P* |
| Aged  Man  Elevated WBC  Elevated PLT  Elevated HGB  Elevated LDH  Elevated BM blasts  Elevated PB blasts  Abnormal karyotype  *TET1/2* mutation  *IDH1/2* mutation  *DNMT3A* mutation  MDS-related genes mutation  *FLT3-TKD* mutation  *GATA2* mutation | 0.994 (0.947, 1.043)  0.495 (0.117, 2.101)  1.009 (0.994, 1.023)  0.997 (0.987, 1.007)  1.009 (0.971, 1.049)  1.000 (0.997, 1.003)  1.019 (0.981, 1.059)  0.997 (0.967, 1.027)  0.671 (0.081, 5.530)  2.266 (0.457, 11.237)  1.468 (0.363, 5.937)  0.768 (0.182, 3.234)  2.413 (0.575, 10.131)  1.537 (0.366, 6.463)  9.944 (2.369, 41.738) | 0.803  0.340  0.231  0.554  0.649  0.876  0.330  0.836  0.711  0.317  0.590  0.719  0.229  0.557  0.002 |  | 8.366 (1.302, 53.748)  25.573 (4.136, 158.124) | 0.025  <0.001 |

Table S7. Univariate and multivariate Cox regression analyses of OS in *NPM1*^mut^ patients (n=91).

Age, WBC count, platelet count, hemoglobin level, LDH level, BM blasts percentages, PB blasts percentage were analyzed as continuous variables. Variables with a *P*-value of less than 0.5 in univariate Cox regression analysis were selected for inclusion in the multivariable Cox regression analysis. The optimal combination of covariates for multivariable Cox regression was determined via a stepwise selection procedure.
