# Supplementary material for: Influence of genetic co‐mutation on chemotherapeutic outcome in NPM1‐mutated and FLT3‐ITD wild‐type AML patients
Source: Cancer Med. 2024 Aug 9;13(15):e70102. doi: 10.1002/cam4.70102 (PMC11316012; doi:10.1002/cam4.70102)
Supplement: Supplementary file 10 — Table S9. [file CAM4-13-e70102-s008.docx]

| Abbreviation | Expansion |
| --- | --- |
| AML  BM  CR/CRi  CHOP-like mutation *DNMT3A*  *FLT3-ITD*  *FLT3-TKD*  FCM  G-CSF  *GATA2*  HSCT  *HOX/MEIS1*  HD-Ara-C  HR  HLA  *IDH*  LAIPs  LDH  MDS-related genes  MRD  MECOM  *NPM1*  *NPM1*^mut^/*FLT3-ITD*^wt^  NGS  NR  OS  PB  RFS  *TET*  WBC | acute myeloid leukemia  bone marrow  complete remission with or without complete blood count recovery  clonal hematopoiesis of oncogenic potential mutation  DNA methyltransferase 3A  internal tandem duplication of the FMS-like tyrosine kinase 3  tyrosine kinase domain of the FMS-like tyrosine kinase 3  Flow Cytometry  granulocyte colony stimulating factor  GATA Binding Protein 2  hematopoietic stem cell transplantation  homeobox/myeloid ecotropic virus insertion site 1  High-Dose Cytarabine  hazard ratio  human leukocyte antigen  isocitrate dehydrogenase  Leukemia-associated immunophenotypes  lactate dehydrogenase  myelodysplastic syndrome related genes  measurable residual disease  *MDS1*-*EVI1* complex  Nucleophosmin 1  *NPM1*-mutated and *FLT3-ITD* wild-type  Next generation sequencing  not reached  overall survival  peripheral blood  relapse-free survival  Ten-eleven translocation  white blood cell |

Table S9. Abbreviations and their corresponding expansions.
